# The mastery lifestyle intervention to reduce biopsychosocial risks for pregnant Latinas and African Americans and their infants: protocol for a randomized controlled trial

**DOI:** 10.1186/s12884-022-05284-9

**Published:** 2022-12-28

**Authors:** R. Jeanne Ruiz, Kristyn Grimes, Elizabeth Spurlock, Angela Stotts, Thomas F. Northrup, Yolanda Villarreal, Robert Suchting, Melissa Cernuch, Liza Rivera, Raymond P. Stowe, Rita H. Pickler

**Affiliations:** 1 Microgen Laboratories LLC, La Marque, Tx USA; 2 The Ohio State University, Columbus, Ohio, USA; 3 Department of Family and Community Medicine, University of Texas Health Science Center at Houston (UTHealth), McGovern Medical School, Houston Tx, USA; 4 Department of Psychiatry and Behavioral Sciences, University of Texas Health Science Center at Houston (UTHealth), Houston, Tx, USA

**Keywords:** Latinas, African Americans, Acceptance and commitment therapy, Problem solving therapy, Psychological outcomes, Infant outcomes, Progesterone, Estriol, Corticotrophin-releasing hormone

## Abstract

**Background:**

Pregnant Mexican Americans (hereafter called Latinas) and Black/African American women are at increased risk for psychological distress, contributing to preterm birth and low birthweight; acculturative stress combined with perceived stress elevates depressive symptoms in Latinas. Based on our prior research using a psychoneuroimmunology framework, we identified psychological and neuroendocrine risk factors as predictors of preterm birth in Latina women that are also identified as risk factors for Black/African American women.

**Methods/design:**

In this prospective, randomized controlled trial with parallel group design we will explore psychosocial, neuroendocrine, and birth outcome effects of the Mastery Lifestyle Intervention (MLI). The MLI is a culturally relevant, manualized, psychosocial, group intervention integrating two cognitive behavioral therapies for both pregnant Latinas and Black/African American women (total *n =* 221). Study inclusion criteria are: women with current pregnancy at 14–20 weeks gestation, ability to read and speak English or Spanish, self-identify as Latina of Mexican heritage or Black/African American, 18–45 years old, born in the US or Mexico, and currently living in the US. Participants must receive Medicaid or other government-supported insurance, and meet screening criteria for anxiety, depressive symptoms, or stress. Participants are randomly assigned to either the intervention (MLI) or usual care group (UCG) in groups of 6–8 participants that occur over 6 consecutive weeks. Data are collected at 3 time points: enrollment (14–20 weeks gestation), following treatment (20–26 weeks), and 6 weeks after treatment (32–36 weeks gestation). Additional outcome, mediating, and moderating data are collected from the electronic health record during pregnancy and at birth. Analyses will primarily use generalized linear mixed modeling (GLMM) to evaluate the relationships between predictors and outcomes.

**Discussion:**

This RCT will test the efficacy of two combined third generation cognitive behavioral therapies (the MLI), given in a group format over 6 sessions, as compared to a usual prenatal care group, for both Latina and African American pregnant women. If efficacious, it may be provided as an adjunct to routine prenatal care and improve mental health, as well as babies being born too small and too soon.

**Trial registration:**

The trial was retrospectively registered at ClinicalTrials.gov. Bethesda (MD): National Library of Medicine. Identifier NCT05012072, Reducing Pregnancy Risks: The Mastery Lifestyle Intervention (MLI); August 19, 2021. The trial is currently recruiting participants.

## Background

A prenatal diagnosis of depression, anxiety or stress worsens the risk for preterm birth (PTB, gestational age < 37 weeks) in Mexican American and Black/African American women [1]. In 2020, the rate of PTB among Black/African American women, hereafter referred to Black women, was 14.4%, about 50% higher than the rate of PTB among White women (9.1%) [2]. Pregnant Mexican American women, the largest subgroup of Hispanic women, hereafter referred to as Latinas, are also at increased risk for psychological distress which leads to adverse birth outcomes such as PTB, and low birthweight (LBW, < 2500 g) [2]. Data also indicate a disparity in the use of mental health services used by Black and Latina women. In a systematic review [3], the amount of mental health service use during pregnancy for Black and Latina women was between 4 and 5%. Black and Latina women are also less likely to receive follow up for treatment [4]. Barriers to use of services include infrequent screening, particularly in clinics caring for predominantly Black and Latina women, as well as healthcare mistrust [5]. The most common treatment for psychosocial distress has been cognitive behavioral therapy (CBT) [3]. Cultural adaptations of CBT programs hold promise to improve clinical outcomes in racial and ethnic minorities.

Evidence indicates that the causes of PTB are multifactorial [6, 7], with many risk factors that are difficult to modify [8]. Psychological factors appear to be important predictors [9] [1, 10, 11] that are potentially modifiable. Reducing psychological risks has had positive effects on some pregnant women [12]. Though there has been some concentration on reducing psychosocial risks in African American women, there has been minimal focus on decreasing psychosocial risks for Latina women. This is problematic as acculturative stress and perceived stress together are known to elevate depressive symptoms in Latina women [13, 14], particularly those who have assimilated into U.S. culture, thereby increasing their risk of PTB [15]. Many care providers do not recognize or diagnose depression during pregnancy. Moreover, even if depressed women are identified, few are treated [16]. This may leave women at increased risk of both PTB and postpartum depression, which has long-term effects for mother and child [17].

Neuroendocrine factors are important for the maternal-fetal response to psychological distress. Research has demonstrated that neuroendocrine pathways mediate the effects of psychosocial factors on health outcomes, i.e., particularly fetal development and infant birth outcomes [18]. In seminal work, Chrousos [19] linked the neuroendocrine system to the stress response and health outcomes. The hypothalamic-pituitary-adrenal (HPA) axis (in pregnancy, the placental-pituitary-adrenal-axis) is one of the major systems involved with the stress response. The main agent of this axis is Corticotropin Releasing Hormone (CRH); placental CRH is identical to hypothalamic CRH both biologically and in its response to stress [20]. Wadhwa [11, 18] states that the placenta can receive, process, and respond to certain stimuli, and thus may act as the central nervous system in the stress response. Placental CRH facilitates communication between the placenta and fetus. Increases of CRH early in pregnancy have predicted the onset of preterm labor in ours and other’s results [20–25]. The chronicity of stress is particularly important in relation to the initiation of early labor [25]. Estrogen and progesterone are other important endocrine factors linked to CRH and related to labor. Increases in estrogen (estriol**,** E3) and decreases in progesterone shift the endocrine balance. Placental CRH stimulates E3 induced changes in the cervix and uterus, part of the initiation of labor [26]. It is important to explore the effects of an intervention to reduce psychosocial distress on some of the key pathways, such as CRH, progesterone, and estriol. In our previous study with Latinas [27], the combination of acculturation, depressive symptoms, progesterone, and E3 predicted PTB, indicating that the combined impact of these factors produced the greatest risk. We propose to a) test a behavioral intervention to reduce psychosocial risks, and b) explore the impact on neuroendocrine factors and infant birth outcomes.

To address gaps related to interventions for Latina and Black women, we developed, and pilot tested the novel Mastery Lifestyle Intervention (MLI). The MLI is a culturally-relevant, manualized psychosocial group intervention that integrates two evidence-based CBT programs – Acceptance and Commitment Therapy (ACT) and Problem-Solving Therapy (PST) [12]. The proposed study is unique in that the investigators are targeting psychological risk factors with the MLI as well as assessing the intervention’s effect on associated neuroendocrine risk factors [20–25]. The MLI is designed to be integrated into regular prenatal care and delivered by a nurse practitioner (NP) or certified nurse midwife (CNM).

The primary aim of the trial is to determine the efficacy of the MLI in pregnant Latina and African American women to decrease depressive symptoms, anxiety, perceived and acculturative stress, and to improve coping, versus usual care (UC), from baseline (14–20 weeks’ gestation) to end-of-treatment (20–26 weeks’ gestation) and at a 6-week follow-up (32–36 weeks’ gestation). Acculturation is a moderator for Latinas, and psychological flexibility is a mediator for all participants. We are specifically intervening prior to maternal biological changes we have observed to occur at 22–24 weeks gestation [27]. Secondary aims are to explore the effect of the MLI on neuroendocrine risk factors of PTB (CRH, progesterone, estriol) versus UC from baseline to end-of treatment and explore the effect of the MLI on infant birth outcomes (gestational age, birthweight, neonatal intensive care unit (NICU) admission).

We hypothesize that participants in the 6-week MLI will have decreased depressive symptoms, anxiety, stress, disengaged coping, and increased active coping compared to UC at end-of-treatment and 6 weeks after completing the intervention. We also hypothesize that the effects of MLI versus UC on depression, anxiety, stress, acculturative stress, and coping will be mediated by psychological flexibility and moderated by acculturation. We further postulate that compared to UC, MLI participants will have significantly lower mean levels of CRH from baseline to end-of-treatment and significantly higher progesterone levels and lower estriol levels (higher progesterone/estriol ratios) from baseline to end-of-treatment. Finally, we hypothesize that compared to UC, infants born to mothers in the MLI group will have a greater mean gestational age at birth, higher mean birth weight, and fewer NICU admissions.

## Methods

### Study design

The study is a parallel-group (MLI versus UC) randomized control trial (RCT) designed to evaluate the efficacy of the 6-week MLI group sessions compared to routine or usual prenatal care, with psychological assessments at pre- and post-intervention, and 6 weeks after the sessions are complete. The results of the psychological assessments (depressive symptoms, anxiety, and stress) are the primary outcomes. The results of biological measures (progesterone, estriol and CRH) are secondary outcomes. The results of infant outcomes (gestational age, birth weight, Neonatal Intensive Care Unit admission) are the tertiary outcomes. The Human Subject Board of Microgen Laboratories has approved the protocol. Table 1 gives a SPIRIT table that gives a participant timeline table.

**Table 1:**
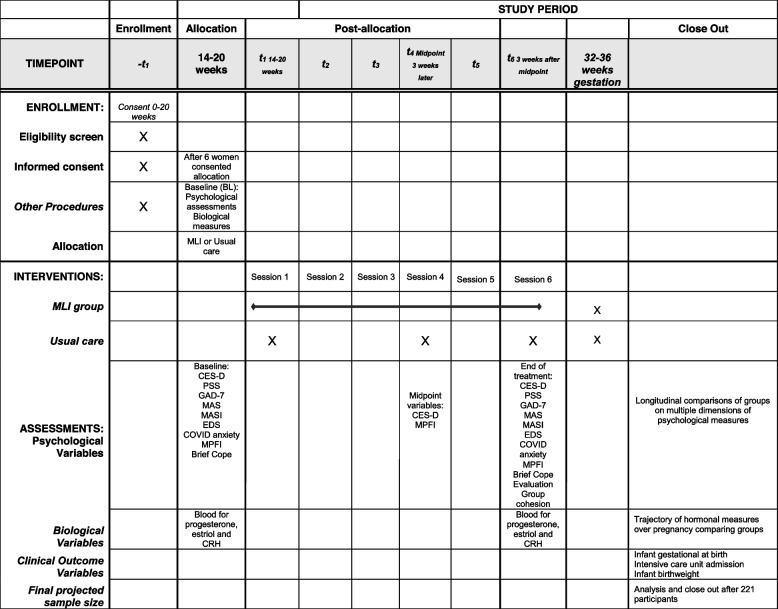
SPIRIT schedule of enrollment, interventions, and assessments

### Participants, eligibility, and screening

We are following participants from a private physician obstetrical practice in Houston Texas in the Texas Medical Center. The practice sees about 40 new pregnant women monthly; 80% are < 10 weeks gestation on their first visit. Inclusion criteria are: a) providing informed consent; b) ability to read and speak English or Spanish; c) singleton intrauterine pregnancy at 14–20 weeks gestation, gestational dating will be reviewed before enrollment via ultrasound administered per standard of care; d) self-identification as Latina of Mexican heritage, or African American; e) age 18 to 45 years; and f) born in Mexico or U.S. born and currently living in the U.S.; g) Medicaid or other government supported insurance; h) women who score ≥ 10 on the Center for Epidemiological Studies of Depression (CES-D) (possibility of mild depression) (< 10 on the CES-D is considered subclinical depression), or ≥ 5 or greater on the Generalized Anxiety Disorder-7 scale (GAD-7) (mild anxiety), or ≥ 14 on the Perceived Stress Scale (PSS) (mild stress); i) willingness to adhere to the MLI regimen or usual care regimen. In our last study using this practice for analysis in Latinas only, 61% of patients had scores on the CES-D ≥ 10 (mild depression), and 30% had scores ≥16 (moderate to severe depression). These cut-off scores were chosen so that the MLI could be evaluated to prevent worsening mental health and detecting possible subclinical depression, as well as a treatment for depressive symptoms, anxiety, or stress.

**Fig. 1:**
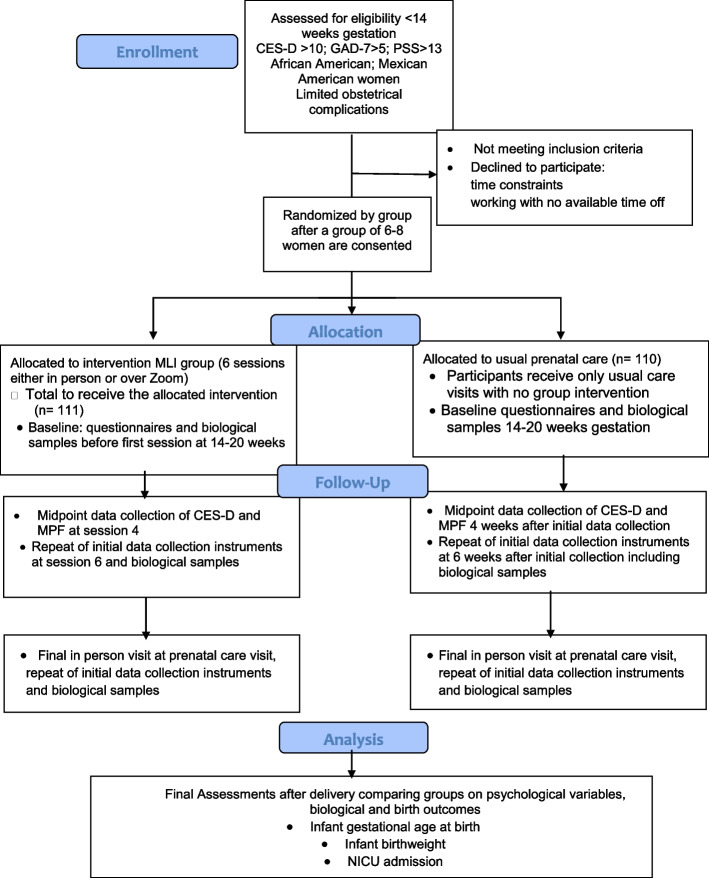
CONSORT flow diagram

Women are excluded after initial review of the electronic health record (EHR) if they have a); major systemic infections such as HIV, hepatitis; b) are < 18 years of age; c) are enrolled in a prenatal program such as the Nurse Family Partnership; d) have severe cognitive or psychiatric impairment per judgment of providers that precludes cooperation with study protocol; e) inability to read English or Spanish; or f) are pregnant with more than one fetus. Women who develop gestational diabetes mellitus (GDM) after enrollment will remain in the study, but Type 1 or 2 diabetes is an exclusion. The investigators will consider development of hypertension or preeclampsia, pyelonephritis, or GDM a modifier in analysis of infant outcomes. Currently taking an antidepressant is not an exclusion. Our proposed sample is 221 women over 5 years, with half receiving the MLI and half usual care (1:1 ratio). See Fig. 1 for a CONSORT flow diagram of the study.

### Recruitment and randomization

We recruit 6 to 8 women per group every 6 weeks with a total of 8 groups per year. The Clinical Research Assistant (CRA) scores the CES-D, GAD-7, and PSS before the potential participant is seen by the physician for their first obstetrical visit. If they have scores above the identified minimum, the CRA then tags the charts for the physician to introduce the study with a brochure. The physician screens the patients for obstetrical exclusions and determines any potential fetal anomalies prior to introducing the study. When he is done with his initial examination, the physician notifies the CRA, and the CRA discusses the study with the patient in a private location. This conversation with the potential participants to thoroughly discuss the benefits of the study and to establish rapport with the participant is important for consent and attrition management. Once written consent is obtained, the participant’s gestation at the first obstetrical visit determines timing of the baseline measures and subsequent randomization.

Participants are randomized in groups. The random assignment of groups was done by our statistician using Random Allocation Rule to two blinded code letters viaR package *randomizeR*. Each code letter was then randomly assigned to one of the two groups by the CRA. Unblinded codes were recorded and saved by the CRA, with retrieval instructions transcribed in case of emergency. Group assignments in opaque envelopes are opened by the CRA after 6–8 women have consented; participants are notified of group assignment by the CRA.

### Attrition management

The CRA also discusses the commitment for the six group sessions (if the participant is randomized to an MLI group) and discusses potential obstacles to attendance and potential solutions. The CRA telephones or texts the participants to stay in touch with them between consent and the time for the baseline, so that the participants know they are valued. The CRA also reminds the participants of weekly group sessions if randomized to an MLI group, and in coordination with the other CRA, plans make-up sessions via teleconferencing for missed sessions, or the need for teleconferencing at a regularly scheduled session if a participant cannot be present for the in-person meeting. The primary investigator, co-investigators, and statistician are blinded to group assignment. The CRAs are unblinded by necessity as they need to follow participants longitudinally to collect data. The NP interventionists know who is in the MLI groups, but they do not collect data or have access to data from either group.

### Intervention

We developed and pilot tested the novel Mastery Lifestyle Intervention (MLI), a culturally-relevant, manualized psychosocial group intervention that integrates two evidence-based CBT programs – Acceptance and Commitment Therapy (ACT) and Problem-Solving Therapy (PST) [12]. The intervention originally was targeted to address psychological risk factors demonstrated to be a factor in adverse pregnancy outcomes in Latinas [28, 29]. It has now been expanded to African American pregnant women, while also measuring the effect on associated neuroendocrine risk factors. We integrated ACT and PST in the MLI as they complement and build on one other. ACT enhances flexibility in responses to internal stimuli (thoughts, feelings, physical sensations), while PST teaches more concrete problem-solving skills in the external environment. Both strategies assist women in pursuing valued goals, teaching effective coping strategies to manage changes that women may face during pregnancy. Mindfulness exercises are included with each session of the MLI. We have developed an MLI facilitator manual and participant handbook for the group sessions. The MLI manuals have been translated into Spanish and back translated into English for Spanish-speaking participants.

The MLI is a 6-week program designed to be integrated into regular prenatal care to facilitate more comprehensive care delivered by a NP or CNM. We have targeted the timing of the MLI prior to maternal biological changes that occurred at 22–24 weeks gestation in our previous work [29]. Ninety-minute sessions were planned for in-person offerings; these occur in a private location adjacent to the physician office where the participants receive prenatal care. Because of COVID-19 restrictions (and patient concerns) and unique patient-related events (e.g., childcare-related conflicts, work conflicts), we also provide some sessions via Zoom; we are carefully recording in-person versus Zoom attendance and monitoring missed sessions to explore these as potential effect modifiers. Women receive the MLI participant handbook in their choice of English or Spanish. The handbook contains space for reflection and activities to complete at home.

### Criteria for discontinuing or modifying intervention

Participants may not be able to continue in the MLI group due to various reasons, such as death in the family, moving to a different provider and city, and/or inability to attend the MLI sessions either virtually or in person. When a subject discontinues from the MLI group but not from the study, the participants will complete the remaining study procedures (i.e., completion of second round of questionnaires and laboratory assessment), and data collection at 32–36 weeks gestation, if at all possible.

If the score on the prescreen CES-D is high (> 35), the physician is notified for follow up and possible referral, relying on his assessment for follow up referral. If a deleterious clinically significant finding is identified (including, but not limited to changes from baseline) after enrollment, the PI Dr. Ruiz or qualified designee (Dr Stotts) will determine if any change in participant management is needed. Any new clinically relevant finding will be reported as an adverse event (AE).

The data to be collected by the CRA at the time of study intervention discontinuation will include the following:

- The reason(s) for discontinuing the participant from the intervention, and methods for determining the need to discontinue.
- If the participant is due to complete assessments within 2 weeks of being discontinued from the study intervention, those assessments will be administered at the time of discontinuation; if the next scheduled assessments are more than 2 weeks from the discontinuation date, the discontinued participant will wait for the next scheduled assessment. Thereafter, the participant will be included in all future scheduled assessments, even though not participating in the intervention. In addition, the birth outcome data will be collected for all participants, once they have been randomized.

### Usual care group (UCG)

The UC group receives regular prenatal care as identified by the American College of Obstetricians (ACOG) with an experienced physician who has been in practice caring for low-income ethnic minorities for many years. If the score on the prescreen CES-D is high (> 35), the physician is notified for follow up and possible referral, otherwise they receive no behavioral therapy during usual care during their pregnancy.

### Measures

Demographic variables include age, marital status, education, insurance type or self-pay, country of birth, years living in the US, primary language, residence in public housing, and number of times the participant has moved within the last year. Data on income, housing, mobility, and type of insurance will be used to assess socioeconomic status.

Obstetric variables include obstetrical history (gravidity, parity, number of miscarriages or induced abortions), last menstrual period, and ultrasound determination of expected date of confinement, height/weight for calculation of body mass index, infections (vaginal or systemic), medications (particularly selective serotonin reuptake inhibitor (SSRI) use or progesterone) other prescription or illegal drug use, and any other medical risk factors for PTB (GDM, preeclampsia, history of PTB, etc.).

Table 2 lists the self-report questionnaires.

**Table 2:** Self-report questionnaires for data analysis

Measure	Purpose; Variable type	Times Measured Baseline =16–20 wks. Time 2 = 20–26 wks. Time 3 = 26–32 wks.	# Items	α	Spanish
MASII Multidimensional Acculturation Scale [30]	Cultural identity; Language proficiency: **Moderator**	Baseline	22	.78–.93	**Yes**
CESD [31]	Depressive symptoms **Primary outcome**	Prescreen, Baseline; Midtreatment (19–23 weeks) for MLI group only. Time 2 and 3	20	.85- .94	**Yes**
GAD-7 [32]	Anxiety; **Primary outcome**	Prescreen, Baseline, Time 2 and 3	7	.89	**Yes**
PSS Perceived Stress Scale [33]	Global measure of stress **Primary outcome**	Prescreen, Baseline, Time 2 and 3	10	.79	**Yes**
MASI Multidimensional Acculturative Stress Inventory [30]	Measure of acculturative stress **Primary outcome**	Baseline, Time 2 and 3	36	.93	**Yes**
The Brief Cope [34]	Positive versus negative coping; **Primary outcome**	Baseline, Time 2 and 3	28	.86–.89	**Yes**
Multidimensional Psychological Flexibility Inventory (MPFI) [35]	Psychological Flexibility **Mediator**	Baseline, Mid-treatment (19–23 weeks), Time 2 and 3	22	.97	**Yes**
Group Climate Questionnaire [36]	Measures if the group is engaged, conflictual, or avoiding interaction; **Covariate**	Time 2 taken by NP and participant	12	.9	N/A
The Everyday Discrimination Scale [37]	Measures day to day discrimination **Covariate**	Baseline,	10	.77	Yes

Biological measures are collected at Time 1 (baseline, 14–20 weeks) and Time 2 (completion of the intervention or control period, 20–26 weeks). Blood is centrifuged and separated into aliquots of plasma per standard protocols used in our previous studies. Blood is stored at − 80 °C. Progesterone and estriol will be run in batches with ELISA. Plasma samples for CRH with Aprotinin 500 IU/ml added will be used to test for CRH by radioimmunoassay (RIA; Phoenix Pharmaceutical Incorporated, Belmont, CA) following our previously established protocol in Dr. Roger Smith’s laboratory in Australia [38]. Urine samples are prescreened for cotinine with 1-Step Rapid Nicotine test for both groups at Times 1 and 2 and then assessed urine cotinine with ELISA for positive results in the laboratory at Microgen.

Infant outcomes  will be obtained from the EHR. Pregnancy duration will be recorded in completed weeks and days. Birthweight will be recorded in grams. We will collect data on any NICU admissions including diagnoses for the infant, especially those linked to pregnancy complications. We will collect data on medically indicated PTB versus spontaneous PTB, and early term births (37–39 weeks). We will use gestational age as a continuous variable as longer gestation, even among term infants, benefits both cognitive and motor development [39]. Infant biological sex will be used in analyses of infant outcomes as male sex is associated with less optimal outcomes [40], maternal prenatal depression and impaired neonatal motor behavior [41], and female sex is associated with prenatal maternal depression and increased anxiety.

### Intervention fidelity

Intervention fidelity is assessed by study co-investigators who are experts in psychological interventions. They monitor and evaluate the fidelity of content delivery for MLI groups conducted in person or over secure teleconferencing. One co-investigator attended the entire first round of group sessions to review fidelity to the intervention, providing feedback to the NP. Thereafter, the co-investigators have evaluated compliance and competence with the protocol via video recording. Compliance with the protocol is assessed using the MLI Fidelity Checklist (adherence scale). The scale addresses the core elements prescribed in the manual. The raters indicate on a scale from 1 (none) to 5 (very much) the extent to which the treatment element was present in the recorded session.

### Data management plan

Data entry of the psychological measurements will be ongoing longitudinally, with every baseline assessment from a participant, follow up 1, and follow up 2. We are using the RedCap data entry system on tablets for each of the time points for the questionnaires in Table 1. After the participant finishes each assessment time point, the CRA submits the data stored on the tablet to the secure, encrypted RedCap database housed at UT Houston McGovern School of Medicine. For the obstetrical data, the CRA collects the data from the obstetrical chart from the initial obstetrical visit out of the participating MDs practice, at 20–26 weeks gestation, and at 32–36 weeks gestation. She enters data onto an Excel spreadsheet on the secure drive. In addition, the CRA obtains data from the physician about the maternal and infant outcomes and enters onto the Excel spreadsheet. The biological results will be determined from plasma in batches of 40 samples at Microgen laboratories, to also be entered on the Excel spreadsheet. The samples are stored in the freezer at the office of the study, and then sent to Microgen laboratories by car when multiple sample boxes are obtained.

The remaining samples that are not used for analysis will be available for use in other future ancillary studies. A data dictionary has been developed for variable names. The one co-investigator is responsible for transferring the data into either SAS, or SPSS statistical programs for analysis. The use of a secure shared drive with multiple authentication systems ensures that the confidentiality of the data is not lost at any time. Only computers approved by UT Houston Institutional Technology department are allowed to access the shared drive.

### Data monitoring plan

The Principal Investigator (Dr Ruiz) is responsible for knowing the policies of the local IRB (Microgen Laboratories IRB). The PI adheres to these policies and maintains accurate documentation of IRB correspondence and reports. The PI is responsible for documentation and handling of all possible study-related adverse events. Staff training, manual-driven processes, and periodic audit of data collection/entry are all part of study monitoring. The PI, Clinical Research Assistants (CRAs), and all investigators adhere closely to the data and safety monitoring plan to ensure participants in the study are protected and to ensure their interests are not made secondary to the interests of the scientific investigation. The investigative team has expertise in the areas of ethics, human subjects’ research, obstetric and pediatric populations, and biological results.

#### The data safety monitoring board (DSMB)

Consists of an obstetrician unrelated to the study, a statistician unrelated to the study, and a health psychologist from an unrelated university. The DSMB is independent from the sponsor, Microgen laboratories.

#### Composition of the DSMB plan

The committee meets annually and more often, if necessary. Specifically, the DSMB performs the following activities: (a) reviews the research protocol and plans for data and safety monitoring; (b) reviews major modifications to the study proposed by the PI prior to implementation; (c) evaluates study progress, including data quality, participant recruitment rates, retention rates, outcome data collection and other trial metrics as stated in the Study Timeline/Milestones. Also reviewed is data related to serious adverse events (SAEs), and an evaluation of risk-versus-benefit profiles. The PI is responsible for communicating information and recommendations to appropriate persons at the Microgen IRB regarding the assessment of issues or problems and effective resolution. In the unlikely event of serious study-related adverse events, the PI, in consultation with the IRB and National Institutes of Child Health and Human Development (NICHD) will consider trial discontinuation and/or termination. The PI, CRAs, and entire investigative team is responsible for protecting the confidentiality of the trial data and the results of monitoring.

#### Adverse events

Adverse Events (AEs) are recorded by study staff on an Adverse Event Log as they occur and will be reported as a summary on an annual basis. Serious AEs (SAEs) will be reported immediately (verbally within 24 hours) to Microgen IRB, all investigators, and to NICHD. A written report should follow as soon as possible but within no more than 3 days. The written report will be in the format required by Microgen IRB and will contain information regarding the date of the SAE, description of the SAE, severity rating (Grade 1 to 4), assessment of cause, whether the SAE indicates an increased risk for current or future subjects, and whether changes to the informed consent form are necessary.

#### Study discontinuation

No interim analyses are planned to evaluate futility or superiority of treatment. A pattern of repeated study-related adverse events (e.g., participant deaths or serious bodily or other harm resulting from participation) will warrant study discontinuation, in consultation with investigators, Microgen IRB, and NICHD/NIH. If it is agreed upon to discontinue the study or if NICHD rescinds funding support, all research stakeholders (including participants) will be advised of the decision and appropriate recourse.

### Statistical methods

#### Power calculation

This study is powered for the primary hypothesis testing the effect of the MLI versus UC on depression, anxiety and stress and was calculated using k = 1000 Monte Carlo simulations under different conditions in SAS 9.4. Specifically, power estimates focus on the detection of a statistically significant interaction between time and treatment group in generalized linear mixed models (GLMM). Table 3 describes power to detect effect sizes as small as those shown given the following: (1) a range of plausible attrition rates (~ 15%, ~ 20%, and ~ 25%) (2) a recruited sample size of *N* = 221 women, (2) a correlation of *r* = .50 between consecutive observations (baseline to week 6, week 6 to week 12), (3) a correlation of *r* = 0.05 for all observations collected at the same time point, and (4) α = .05/5 = .01. In practice, the statistical significance will rely on the false discovery rate to adjust for multiplicity; this will rely on the observed *p* values. The powered effect sizes above align with those found in previous research and pilot studies. Our pilot study (*n* = 15) showed an effect size on anxiety that ranged from d = −.37 to d = − 1.5; for depression, the effect size ranged from d = −.45 to d = −.76. Our results align with other studies using ACT. McCracken [42] used the Patient Health Questionnaire-9 in a RCT (*n* = 73) to measure depression pre- and post-treatment after ACT for chronic pain and found a Cohens *d* = .58. Majumdar [43] found a medium effect for group ACT (n^2^ = .07, or d = .55).

**Table 3:** Sample Size and Power

Sample Size	15% Attrition | *N* = 200 (100/group)		20% Attrition | *N* = 190 (95/group)				25% Attrition | *N* = 180 (90/group)		
**Power**	81%	86%	91%	78%	84%	90%	75%	82%	88%
**Effect Size**	0.3	0.3	0.3	0.3	0.3	0.3	0.3	0.3	0.3

### Analysis

All variables will be summarized with descriptive statistics. Categorical data will be summarized by frequency and percent, and continuous data will be summarized by measures of central tendency (i.e., mean/median) and dispersion (i.e., standard deviation). Inferential tests will rely on the statistical significance criterion *α* = .05 (two-tailed). Hypothesis tests with values *p* ≤ .05 will be considered significant. Covariates may potentially be included in statistical models. Modeling assumptions will be evaluated via inspection of residual plots and formal statistical tests. Violations of assumptions will be addressed as needed via by transformation, robust estimation coefficient scaling, stratification, or model respecification. Any hypothesis tests that are unable to satisfy statistical modeling assumptions will be reevaluated via equivalent non-parametric tests if possible.

Analyses will primarily rely on generalized linear mixed modeling (GLMM) to evaluate the relationships between predictors and outcomes. This flexible approach allows modeling normally or non-normally distributed outcomes (specified by distribution family and link function), longitudinal or multilevel data (via inclusion of level-2 terms for participant id), and/or nonlinear effects (via inclusion of spline or polynomial terms).

Longitudinal analyses will model each outcome as a function of the treatment group, time, and interaction between the treatment group and time. If the interaction term is not significant, the model will be reduced to main effects only. Follow-up tests of simple effects will evaluate changes in each outcome over time within treatment groups. Cross-sectional models at each time point will use GLMM to model each outcome as a function of the treatment group. In all models, other types of multilevel data (e.g., participants within groups) will utilize a random intercept for group assignment.

Interaction effects will be described as significant or non-significant. If significant, results then focus on simple effects of time within each group; if non-significant, results focus on models of main effects without the interaction. These results will be presented as parameter estimates with 95% confidence intervals for all models that only measure main effects or within-group models of simple effects in the presence of a significant interaction. Graphical presentation will provide a plot of estimated marginal means of outcomes over time by group.

Single and multiple mediator models will investigate the indirect effects of treatment separately on 6-week follow-up measures of depression, anxiety, stress, and coping via intervening (end-of-treatment) measures of psychological flexibility and acculturation. Mediation analyses will rely on Structural Equation Modeling/Path Analysis using MPlus (v. 8.4) to evaluate direct and indirect effects of treatment on outcomes. In the context of the direct effects of treatment, mediational modeling permits estimates of the indirect effects via intervening variables using the product coefficient method. Ninety-five percent confidence intervals will be constructed using a bootstrap resampling approach.

Prior to hypothesis testing, we will inspect relationships between baseline and demographic variables, treatment groups, and outcomes using GLMM. Baseline/demographic covariates demonstrating a relationship with both predictors and outcomes meet criteria as potential confounders and will result in two models, one with and another without covariate adjustment. If inferences are affected via adjustment, we report both models; otherwise, we will report the less complex model [44], [45].

Some specific covariates to be examined as potential confounders include age, pre-pregnancy body mass index, gravidity, positive cotinine, positive drug screens, fetal sex, group climate scores for MLI sessions, concurrent behavioral therapy, acculturation scores, country of birth, SSRI or progesterone use, obstetrical variables (i.e., preeclampsia, gestational diabetes, history of preterm birth, vaginal or systemic infections during the periods between/during data collection and delivery), and frequency of intervention attendance. For the MLI group, number, and type (phone for missed sessions only vs. in-person) of sessions will be investigated as moderating (i.e., interacting) dose-response effects of treatment.

## Discussion

The study is significant, targeting vulnerable minorities at risk for maternal and infant morbidity, mortality, and health care costs. In Houston, Texas, where this study occurs, 1 in 9 babies are born preterm with PTB rates of 10.9% for Latinas and 16.4% for Blacks [46], respectively. If shown to be effective in increasing gestational age, the MLI holds promise to reduce the morbidity and mortality associated with high rates of PTB. The intervention may also advance healthcare as it could be integrated into standard prenatal care, thereby, improving outcomes for many pregnant women. The results of this trial will be disseminated in appropriate journals and presented at professional meetings. If successful, results will be presented to administrators who supervise Medicaid in the United States, as a potential program to improve maternal and infant health along with prenatal care.

This rigorous RCT (with blinding and comparison of multiple outcomes) is being conducted in a real-world obstetric setting with ample resources available nearby from the Texas Medical Center and as such, has high generalizability. The investigators will examine multiple emotional distress outcomes to determine which is the most affected by the intervention. In addition, the study and protocol may empirically show differences in the endocrine profile related to PTB (progesterone, estriol, CRH), a potential confirmation of effectiveness. The interdisciplinary investigators have varied expertise in obstetrical nursing, pediatric nursing, endocrinology and immunology, clinical psychology, social work, and biostatistics. The academic center environment, a biobehavioral laboratory, and a large clinical practice are conducive to high-quality research. There are plans for future work to expand to other groups with a high PTB rate and to the postpartum period, a time of vulnerability for continued emotional distress.

## Data Availability

The data from this study is restricted until the research is finished, and the database will remain locked until the completion of the study. The researchers will have private access to the data until publications are completed from the work. Once the publications are completed, the database will be open to other researchers if requested. The database will be stored at Microgen Laboratories as well as with the PI, Dr. Ruiz. Professor R. Jeanne Ruiz may be contacted after the year 2025 when the study is complete, for requests related to obtaining a copy of the database: jruiz@microgenlabs.com.

## Abbreviations

HIV: Human Immunodeficiency Virus
NP: Nurse Practitioner
CNM: Certified Nurse Midwife;
PI: Primary Investigator
ELISA: Enzyme Linked Immunosorbent Assay
